# A Novel Eddy Current Testing Error Compensation Technique Based on Mamdani-Type Fuzzy Coupled Differential and Absolute Probes

**DOI:** 10.3390/s18072108

**Published:** 2018-06-30

**Authors:** Ahmed N. Abdalla, Kharudin Ali, Johnny K. S. Paw, Damhuji Rifai, Moneer A. Faraj

**Affiliations:** 1 Faculty of Electronic and Information Engineering, Huaiyin Institute of Technology, Huaian 223003, China; 2 Faculty of Electrical and Automation Engineering Technology, TATI University College, Kemaman District 24100, Malaysia; kharudin@tatiuc.edu.my (K.A.); damhuji@tatiuc.edu.my (D.R.); 3 Institute of Sustainable Energy (ISE), UNITEN, Selangor 43000, Malaysia; Johnnykoh@uniten.edu.my; 4 Electrical and Electronics Department, Faculty of Engineering, Omar Al-Mukhtar University, Al Bayda P.O. Box 991, Libya; Mod8491@gmail.com

**Keywords:** lift-off, ECT, fuzzy logic, absolute probe, differential probe

## Abstract

Eddy current testing (ECT) is an accurate, widely used and well-understood inspection technique, particularly in the aircraft and nuclear industries. The coating thickness or lift-off will influence the measurement of defect depth on pipes or plates. It will be an uncertain decision condition whether the defects on a workpiece are cracks or scratches. This problem can lead to the occurrence of pipe leakages, besides causing the degradation of a company’s productivity and most importantly risking the safety of workers. In this paper, a novel eddy current testing error compensation technique based on Mamdani-type fuzzy coupled differential and absolute probes was proposed. The general descriptions of the proposed ECT technique include details of the system design, intelligent fuzzy logic design and Simulink block development design. The detailed description of the proposed probe selection, design and instrumentation of the error compensation of eddy current testing (ECECT) along with the absolute probe and differential probe relevant to the present research work are presented. The ECECT simulation and hardware design are proposed, using the fuzzy logic technique for the development of the new methodology. The depths of the defect coefficients of the probe’s lift-off caused by the coating thickness were measured by using a designed setup. In this result, the ECECT gives an optimum correction for the lift-off, in which the reduction of error is only within 0.1% of its all-out value. Finally, the ECECT is used to measure lift-off in a range of approximately 1 mm to 5 mm, and the performance of the proposed method in non-linear cracks is assessed.

## 1. Introduction

Nondestructive evaluation (NDE) deals with the inspection of an object, determining its properties without destroying its usefulness. The application of eddy current is widespread for measuring electrical conductivity, for defect identification on metallic material and for assessing coating thickness on pipes. Various parameters were considered for the testing methods, which include the type of signal, the amplitude of the signal and the phase angle of the probe, where each of these parameters will affect the signal whenever there is a crack in the plate or workpiece surface [1,2,3]. ECT is also used as a quality control tool in various industries to detect a defect and inspect the condition of samples [4,5,6]. The condition of samples may be related to the surface cracks, sub-surface flaws [3,7,8] and degradation of samples. The eddy current method is also able to measure the thickness of coatings or paint, not only in micrometers but also up to millimeters [9].

Much progress has been reported in the literature, causing eddy current testing to become more attractive in relation to quality control and inspection fields using a pulsed eddy current signal [10,11,12,13]. The probe style and check parameters should be chosen based on a deep understanding of the flaw that the technique is applied to for investigation purposes [14]. Signal EC is highly effective in reducing the lift-off to 2.16% according to the thickness of the plate [15]. High sensitivity for defect measuring and developing eddy current detectors for duplexes requires a specified sampling surface for measuring and detecting defects. Lu et al. [16] proposed a zero-crossing frequency index to reduce the effect of lift-off for magnetic plates. This modified index is a mathematical manipulation derived from the relation between the permeability and zero-crossing frequency from Dodd and Deeds’ method. The proposed index was verified by both simulation and experimental data, and the permeability error reduced within 7.5%, which was caused by liftoff. Lu et al. [17] proposed a novel index compensated peak frequency that is linked to the thickness which is virtually independent of lift-off. This index can be obtained from the measured multifrequency inductance spectral data. The experimental data of thickness measurements proved the accuracy of this approach at different lift-offs to be within 2%.

Many researchers have investigated the advantages of using artificial intelligence in ECT. Rosado et al. [18] proposed nonlinear regressions and artificial neural networks (ANNs) to estimate the parameters of defects tested using an eddy current probe. The ANN was predicted to deflect overfitting, but the comparison between linear and nonlinear regressions are not discussed. Guohou et al. [19] investigated a multi-sensor application for data fusion in defect evaluation. The defect parameters and conductive material are refined by using the theory of Dempster–Shafer in fuzzy inference. The fuzzy set theory was used in calculating the BPA values in the D-S theory, and then the D-S rule of combination is applied to fuse the data from UT and ECT, but the frequencies used are not discussed in detail for this research. Shejuan et al. [8] investigated the function of pulsed eddy current testing (PECT) signals in the sizing of wall thinning defects. The normalized difference of PECT responses significantly reduces the influence of variation in magnetic permeability; nevertheless, a microstructural investigation is needed to confirm this hypothesis [10]. Habibalahi et al. [20] studied pulse eddy current testing for stress accuracy measurement using ANN. The evaluation of stresses and residual stresses, which are the key factors in mechanical component performance, has been a big challenge, but the technique used is not applicable in measuring stress, even in aluminum. He et al. [21] proposed PEC defect automated classification in aircraft multi-ply structures with interlayer gaps and lift-offs. The PEC technique for defect automated classification can effectively eliminate the air-gap and lift-off effect in multi-layer structures. However, the limitations of this work are regarding the calculation time of the three optimized SVM methods, which have not been compared. D’Angelo & Rampone [22] investigated the effectiveness of the neural networks approach for shape defect classification. The classification based on feature vectors split the image into three geometric parameters: the length, width, and orientation angle of the defect. Buck et al. [23] investigated the complex relation between the inspection data and defect properties by using ANNs. PECT feature extraction and deterministic ANNs have been combined to estimate four separate experimental parameters, but the comparisons between PEC and coil probe are not discussed in this research. Therefore, the combination of hardware and software, which includes the intelligent technique, should be carried out to ensure that the accuracy of the data obtained is high with regard to the lift-off and error compensation.

In this paper, a novel eddy current testing error compensation technique based on fuzzy coupled differential and absolute probes was proposed. The proposed probe consists of three coils which are used as follows: two coils work as a differential probe and one coil works as an absolute probe. Both of the probes use the same AC excitation signal source. The input membership functions (MF) were chosen based on the simplicity of the coding algorithm for hardware-based real-time implementation. For each MF, the number of MFs besides the fuzzy rules should be built up according to their dissimilar characteristic groups. Inaccuracy and error analysis has been done, and an error compensation method for lift-off has been proposed by using a Mamdani-type fuzzy inference system. In order to demonstrate the measurement error of the sensor under study caused by the lift-off variations, an experimental setup for data collection has been conducted. The dissimilar frequencies are implemented to test cracks with dissimilar depths and shapes.

## 2. Related Work

### 2.1. Lift-Off in ECT

Eddy current testing is strongly affected by the amount of lift-off, which is defined as the separation distance between the excitation coil surface and the conducting material surface. This distance changes the mutual inductance of the circuits; as lift-off increases, the amplitude of the eddy current induced EMF in the secondary coil decreases, which can result in the misinterpretation of signals as flaws. At significant lift-off, no detectable EMF will be induced in the secondary coil due to the sample [24,25,26].

Compensation algorithms are developed based on the data collected from the ferromagnetic media (FEM) model [27]. The basic idea is to compensate for various experimental parameters that affect the measurement from the receiver in the transmit–receive probe. Compensation algorithms can be developed to compensate for various factors such as probe velocity, the origin of sampling, permeability and lift-off. Reference [12] presents various invariant techniques and transformations for lift-off compensation. An invariant represents a quantity which is definite to an object. It must be invariant under any choice of coordinate system. As an example of a transformation, consider a coordinate system *x*, *y* in Cartesian coordinates. Let *p*(*x*, *y*) be a point in this coordinate system. Consider another coordinate system, X’Y’, which is translated by a factor (*x_a_*, *y_a_*) and rotated by an angle. The transformation of *p* into *x’*, *y’* is given by

(1) $$x^{\prime }=xcos\theta -ysin\theta +x_{a}$$

(2) $$y^{\prime }=xsin\theta +ycos\theta +\;y_{a}$$

Equations (1) and (2) describe the property of a point in another coordinate axis. In general, they represent a characteristic of an object in another domain. 

The Euclidian distance between the two points remains unchanged irrespective of the coordinate axes [12]. A similar idea is applied to develop compensation algorithms to compensate for lift-off in array probe eddy current data. This study investigates the use of error compensation, as well as fuzzy logic, for achieving lift-off compensation. In addition, the use of the absolute and differential probe for compensation is also investigated.

### 2.2. Air-Coils for ECT Measuring Lift-Off

Important parameters, such as signal, frequency, lift-off effect and probe direction, are analyzed by using a numerical simulation method, and sample experiments are carried out for verification as the lift-off effect is one of the most important parameters in eddy current testing. Generally, small lift-offs would cause a great impedance (*Z*) change. In [28,29], eddy-current measurements of metallic plate thickness are studied, and the main focus of investigation is the error caused by lift-off variation.

**Figure 1:**
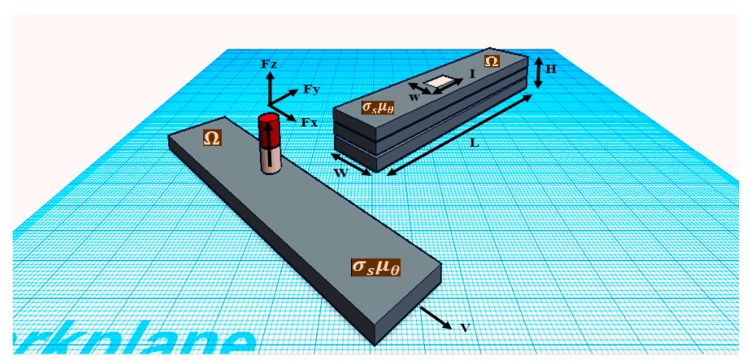
Lift-off Eddy Current Testing basic principle.

Figure 1 illustrates the general principle of ECT, where the relation between the lift-off distance of the permanent magnet and the conductivity of the specimen, magnetic remanence and the statistics of the velocity are determined experimentally. The induced eddy current is due to the relative motion between a permanent magnet and the testing object. Then, based on the Lorents law, the magnetic force will be measured and used to analyze the quality of the specimen. In [28], alternative sensor systems such as pick-up coils are studied, and giant magneto-resistors or Hall probes are used to determine the magnetic field variations.

There are methods for lift-off compensation where eddy currents are used to detect cracks; hence, lift-off becomes an undesired variable. Table 1 includes some previous studies that considered the lift-off issue.

### 2.3. Mamdani-Type Fuzzy vs. Sugeno-Type Fuzzy

The main two fuzzy inference methods are Takagi–Sugeno–Kang and Mamdani’s fuzzy method. Reference [36] shows the difference of fuzzy input selection for both Sugeno-type FIS and Mamdani-type FIS, which resides in the way the crisp output is generated. In [37], Sugeno-type FIS uses a weighted average to compute the crisp output; thus, the output membership functions are either linear or constant. Mamdani-type FIS uses the technique of defuzzification of a fuzzy output. The output from Mamdani FIS can easily be transformed into a linguistic form as the inference result before defuzzification [38]. Therefore, Mamdani’s inference anticipates the output membership functions to be fuzzy sets which are appropriate for comparing the output.

## 3. Methodology

### 3.1. Architecture of the Proposed Error Compensation Eddy Current Testing (ECECT) System

**Figure 2:**
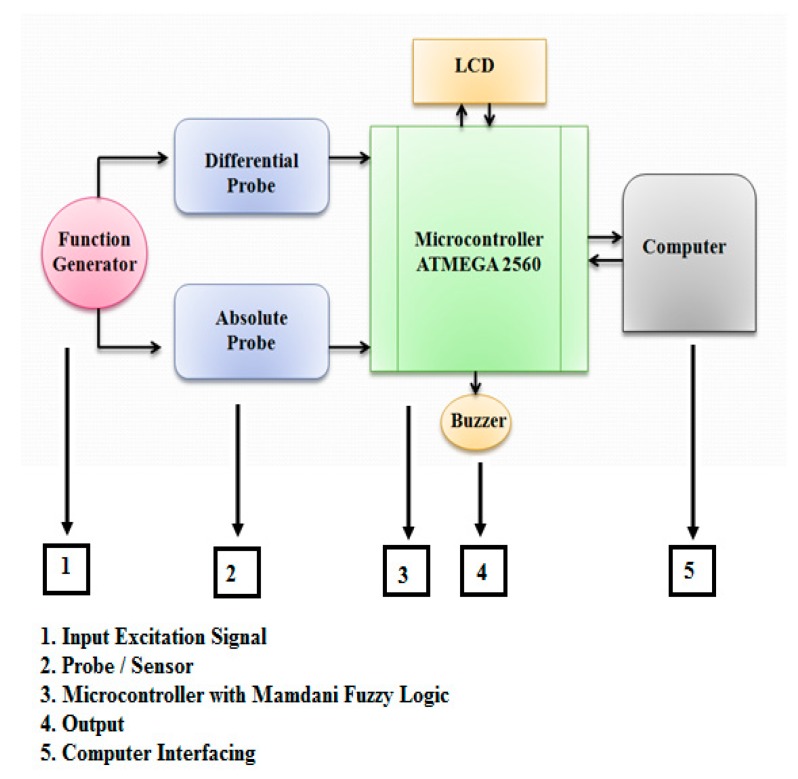
Proposed Architecture of Error Compensation Eddy Current Testing (ECECT) System.

The proposed ECT probe is an important part of the system development in ECECT system design. The architecture design is divided into three main parts, as shown in Figure 2. The first part is the AC excitation signal being chosen as a source supply by using a function generator. In the second part, two input devices are used: a differential probe and an absolute probe. Both of the two input devices use the same AC excitation signal source. The third part is a few controlling or processing devices using Mamdani-type fuzzy logic. 

### 3.2. Proposed Probe Design

**Figure 3:**
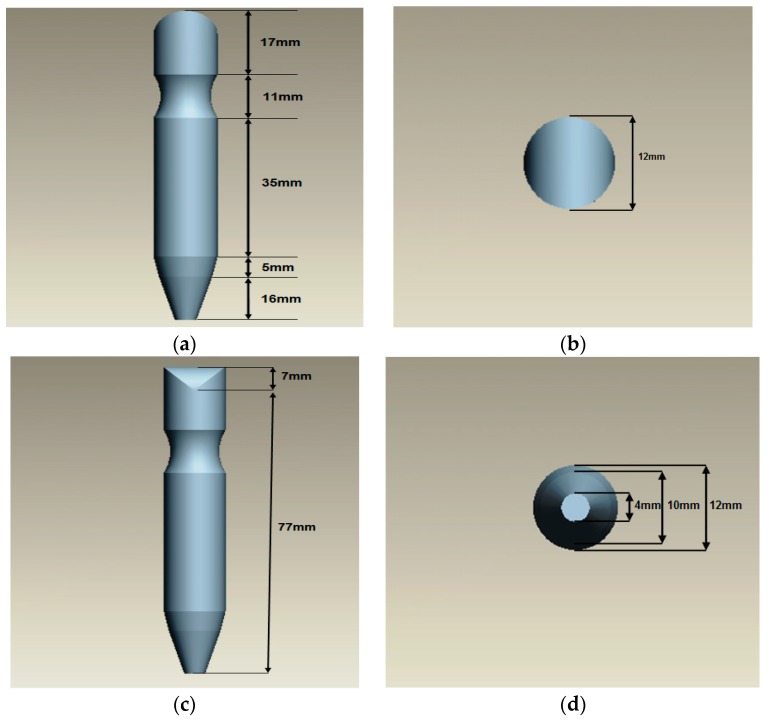
The views of Eddy Current Probe: (**a**) Front View; (**b**) Top View; (**c**) Side View; (**d**) Bottom View.

Probe size design is crucial to ensuring that the probe shoe is appropriate with the size of the probe. From here, the design of the probe (both differential and absolute coils) will have the same size and diameter. The design of the probe follows the actual size of probe; that is, 84 mm in height and 12 mm in diameter. According to the dimension of the probe, the direction view could be shown in four orientations, namely front view, top view, side view and bottom view. Hence, the correct surface, different diameter, different curve and angle of the probe will be determined. Figure 3 shows the dimension of the proposed eddy current probe.

### 3.3. Proposed Mamdani Fuzzy Logic Method in ECT Measurement

**Figure 4:**
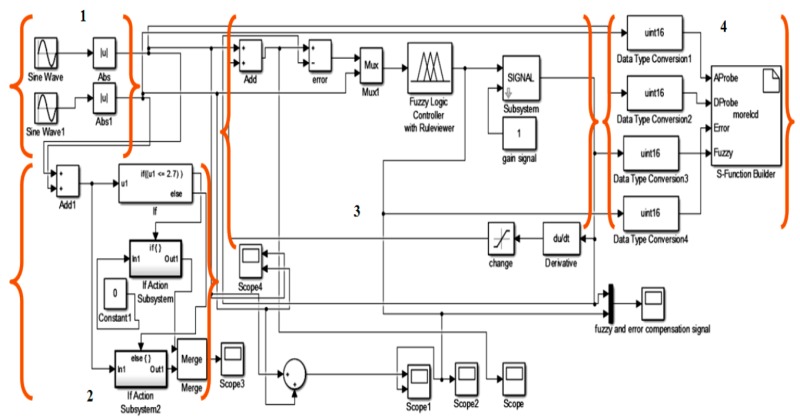
The Simulink Block Diagram Model for ECECT. (1) Input source, (2) Conditioning process, (3) Fuzzy logic process and feedback, (4) Output display.

Fuzzy logic analysis is applied as an administrator to provide the crack data, which is supported with reduced features: amplitude, phase and width. This fuzzy-based decision scheme contains system input, system output, membership functions (MF) and IF-THEN fuzzy rules. The inputs are the characteristics of the crack-specified amplitude, phase, and loop width. The outputs of the scheme are the real crack data specifying depth, width and shape. As shown in Figure 4, each input is an associated fuzzy set, and each fuzzy set accepts its agreed MF. The MF reacts to the degree of each fuzzy set as a member in the membership in the scale of 0 to 1. The fuzzification is executed appropriately to complement a fuzzy set with MFs. Fuzzy rules are declared in IF-THEN lingual condemnations, which describe the relationship between the input and output: for example, IF the amplitude (input) is high, THEN the crack depth is deep (output). Eventually, when more than one fuzzy rule has been applied, and the execution result is lingual (deep), a defuzzification action is required to convey the lingual variables into mathematical crisp values. 

**Figure 5:**
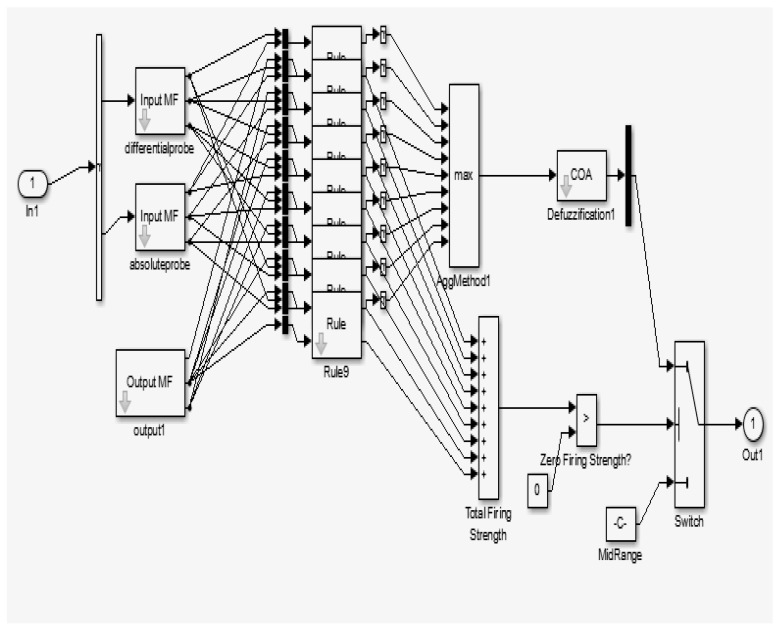
Basic block internal function in Fuzzy Logic.

In the software part, the MATLAB/ Simulink is used to process the probe input signals with the fuzzy output result signal. According to Figure 4 and Figure 5, the simulation block diagram model for ECECT, there are four important parts which are used for the fuzzy logic system. The first part is probe input signals (forming the differential and absolute coils), second is the conditioning process (pretreatment process), and third is the fuzzy logic process and feedback, and lastly output display.

#### 3.3.1. Rules of Fuzzy Logic

To complete the fuzzy logic block setting, the rule of the fuzzy block is set according to the rule editor for lift-off and depth measuring blocks. Table 2 shows the nine rules which have been set based on the Mamdani-type fuzzy rule algorithm.

Lowdefect, depthdefect and dangerdefect are the membership function in fuzzy logic: lowdefect being a setting range of 0–1.75 mm defect, and this defect represents plate scratches; depthdefect being a setting range of 1.76–3.50 mm; and lastly, dangerdefect is a range of 3.51–5.00 mm.

#### 3.3.2. Surface Viewer for Fuzzy Logic

**Figure 6:**
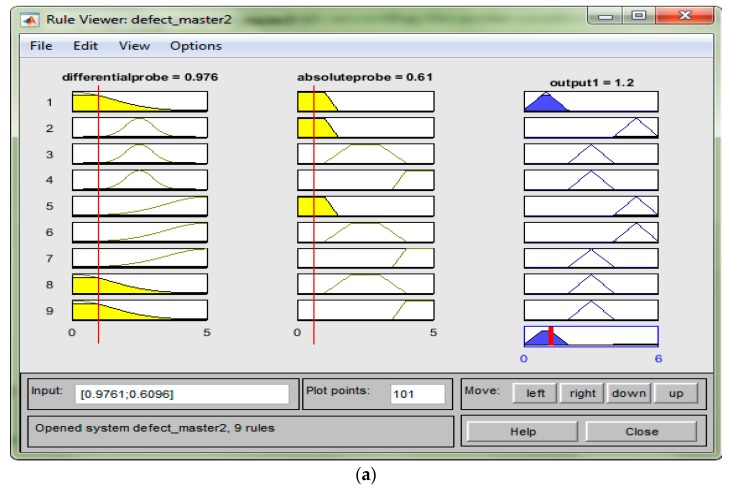

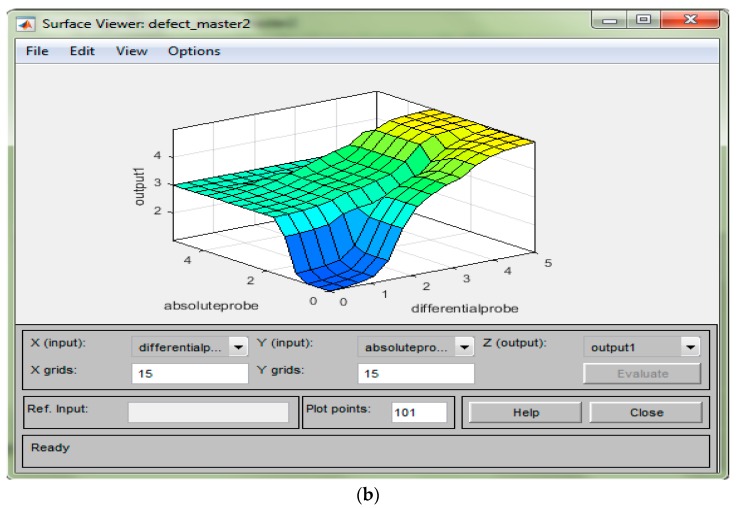
(**a**) Rule viewer; (**b**) Surface viewer.

The fifth step in the fuzzy logic setting is the result of the fuzzy setting by looking at the rule viewer and surface viewer. From here, the user could see the input changes and affect the output for the rule viewer and for the surface viewer shown in the 3D graph. Figure 6a shows the rule viewer for lift-off and the depth of a defect, and Figure 6b is the surface viewer graph. 

## 4. Experimental Setup 

**Figure 7:**
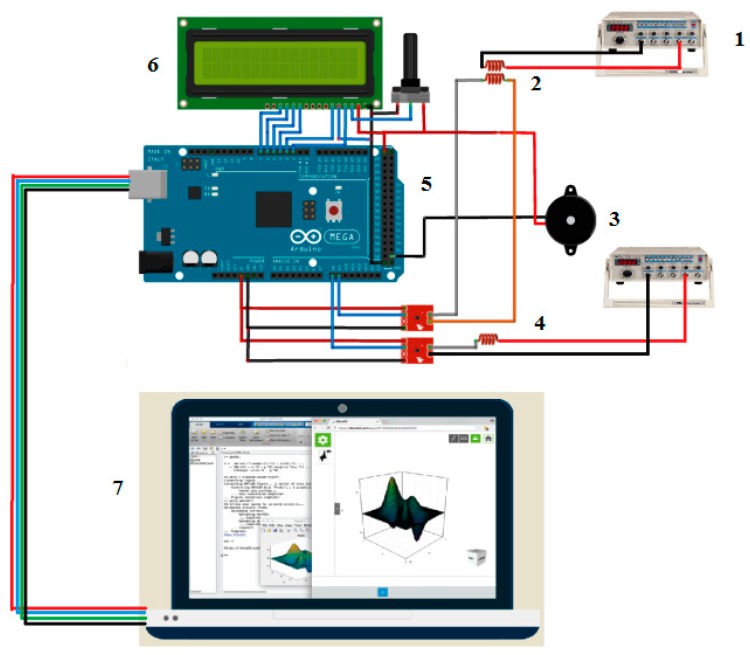
Designing of ECECT Drive Circuit.1: AC signal; 2: Absolute Probe; 3: Buzzer; 4: Differential Probe; 5: ATMEGA 2560 microcontroller; 6: LCD Display; 7: Computer.

Figure 7 shows the schematic circuit; there are five important parts that are essential to ensuring the system’s proper functioning. The first part is the AC supplies that provide the AC voltage for the absolute probe and differential probes. Here, the function generator was used in accordance with the frequency selection and setting. The second part is probe application. The types of probes used are an absolute probe and differential probe. The concept probe is an air-coil probe sensor that generates a magnetic field when the AC supply passes through it. Each defect, especially the crack on the workpiece, occurs then the signal reading shows a different value. The third part is the output devices that function as an indicator and display the depth of the defect on the workpiece. The last part is PC interfacing. MATLAB 2015 is used for signal analysis and for intelligent application on this project. Hence, the filtering signal, fuzzy logic application and output waveform of each defect will be shown on the monitor. The connection between the PC and microcontroller uses a serial communication interface. 

**Figure 8:**
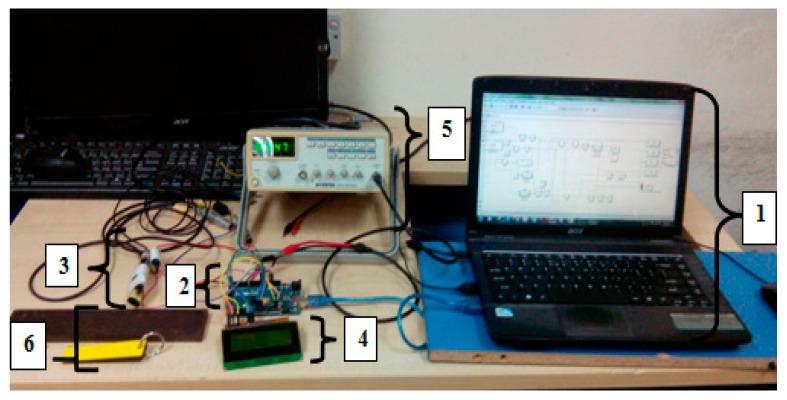
SFECT System Components Setup. 1: Computer; 2: ATMEGA 2560 microcontroller; 3: Absolute and Differential probe combining; 4: LCD display; 5: Function generator; 6: Calibration block.

Figure 8 shows the SFECT system device setup for inspection testing. In SFECT, full system device development has six main parts, in which the first main part is the fuzzy logic interfacing system. In this part, the system of fuzzy logic was developed by using MATLAB Simulink software. After the first part finishes, then the second part for interfacing is developed. Then, the ARDUINO MEGA 2560 was used as a controller device in data input processing and output display. The third part is an input device. Here, the absolute and differential probes are used as a sensor for measuring the defect. The output display for SFECT is an LCD display, where the depth of defect, the thickness of the coating, the percentage of error and fuzzy output value will be displayed on LCD. The function generator is used as a supply or excitation signal for the differential and absolute probe sensor, and lastly the testing is done by using the calibration block to ensure the depths of the defects displayed are correct.

## 5. Experiments and Results

The effectiveness of the lift-off compensation method was measured by comparing the error reduction for uncompensated and compensated methods based on the percentage of error in both simulation and real implementation. In this research, dissimilar frequencies are implemented to test cracks with dissimilar depths and shapes. For each MF, the numbers of MFs besides the fuzzy rules should be built up severally according to dissimilar characteristic groups. In this work, ANFIS in Matlab is applied as a scheme acquiring technique to find the fuzzy logic system. Thus, these trained fuzzy logic engines are implemented to predict the crack data, supporting the extracted features or the combination of the characteristics.

### 5.1. Industrial Probe Measurement Result

**Figure 9:**
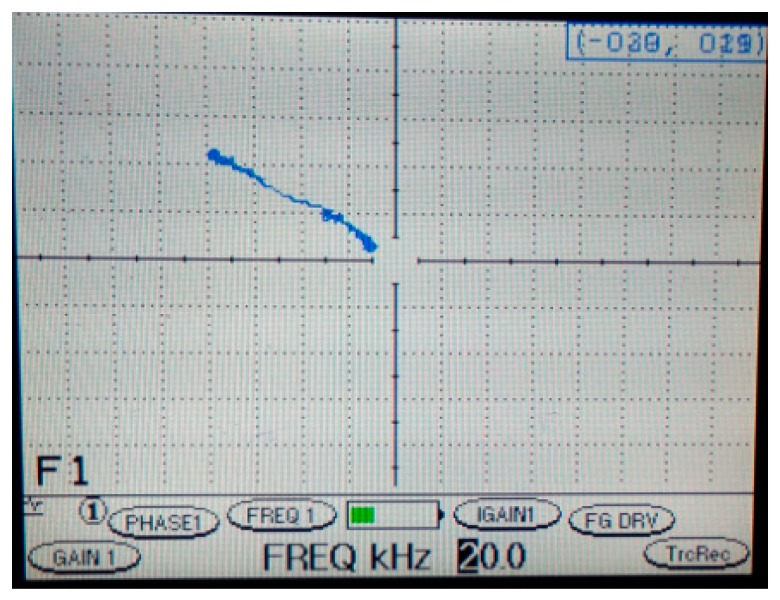
Commercial differential probe Measuring.

In industrial probe measurement, the absolute and differential probes are used. Moreover, the frequencies of both sensors are set at 4 kHz, 10 kHz and 20 kHz. The defect and coating thicknesses were measured by using a calibration block where the depths of the defects are 1 mm, 2 mm and 3 mm and the coating thicknesses are 0.5 mm, 1 mm, 1.5 mm and 2 mm. Figure 9 shows a sample of the graph display according to the depth of defect and coating thickness. This figure refers to the measuring by using a commercial differential probe tester with a frequency of 20 kHz. It is difficult to immediately know the actual depth of a defect because it is necessary to use a calibration block to calculate and compare the base on the table for defect measuring. Table 3 shows the higher amplitude signal according to the depth of defect and coating thickness. Also, the amplitude signal will be high when the depth of defect and frequency setting is high for the differential probe, and on the contrary when coating thickness is low and frequency is high for the absolute probe.

### 5.2. Hybrid Differential and Absolute Probe

**Figure 10:**
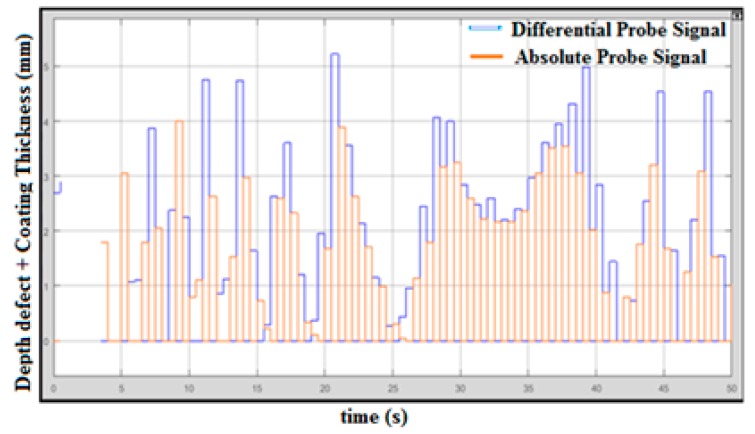
Integrated Probe Output without Coating Thickness.

According to Figure 10, the maximum signal displayed is 5.3 mm, which is less than 10% tolerance. The differential and absolute probe signals are integrated to obtain the right value of the defect. In this testing, however, the coating shield was not used, but the lift-off still occurs when the probes are lifted from the sampling plate testing. This happened because of the resistance of the plate and because the surface plate is not exactly flat. However, the minimum defect measured was lower than 0.5 mm when no defect was present at the surface plate. 

**Figure 11:**
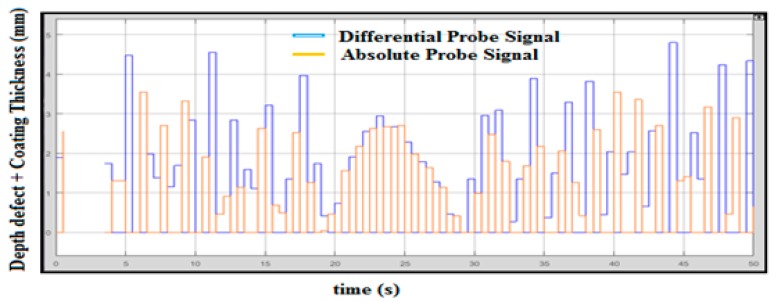
Integrated Probe Output with Coating Thickness.

The plate with coating thickness will affect the measurement of the defect caused by the resistance of coating itself. The rate of frequency for traveling at the plate or pipe will be reduced and the defect measurement reading will be impaired. To reduce the resistance of coating in measurement, the utilization of an absolute probe is hence useful. Figure 11 shows the graph of integration between the absolute probe and differential probe in measuring the thickness of the coated pipe or plate. The maximum defect value shown below is 4.7 mm. The blue signal represents coating thickness and orange represents the depth of the defect. The thickness of the coating is not uniform throughout the whole surface area.

### 5.3. Hybrid Differential/Absolute Probe with Fuzzy Logic

**Figure 12:**
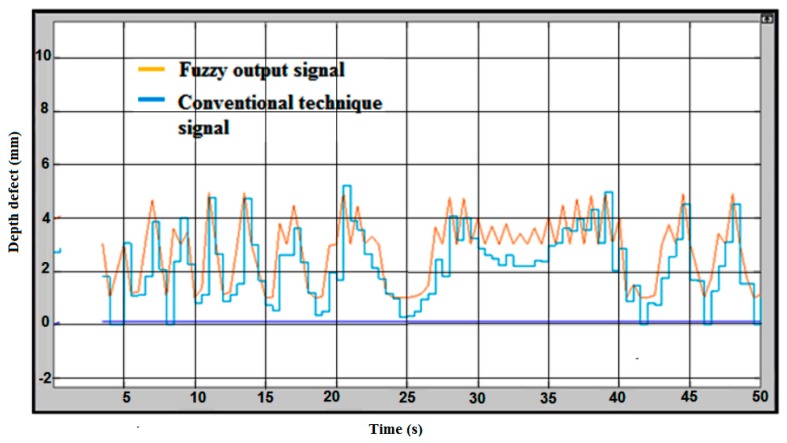
Fuzzy Logic for Error Compensation Signal Output without Coating Thickness.

The intelligence in defect measuring was purposely developed to measure the error compensation. The lift-off compensation frequently occurs in the measurement process. Therefore, the right value of a crack will be defined by considering the lift-off compensation. Figure 12 shows the fuzzy logic for the error compensation of the signal output without coating thickness/lift-off. The handling error is considered as lift-off in the inspection process. The blue line on the graph is considered without fuzzy logic as a conventional technique value, and a yellow line on the graph is a fuzzy output after considering the lift-off for error compensation. The maximum signal for conventional value is 5.3 mm and the fuzzy value is 5 mm.

**Figure 13:**
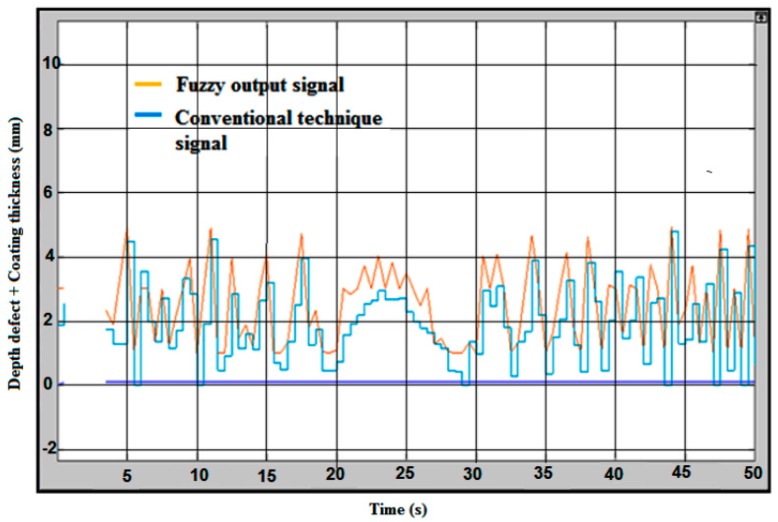
Fuzzy Logic for Error Compensation Signal Output with Coating Thickness.

The difference between the coated and uncoated plate could be seen according to the signal produced during the inspection run. The conventional technique signals are slightly decreased from the actual value of the defect. The effect of the coated plate is shown in Figure 13, indicating the fuzzy logic for the error compensation of the signal output with coating thickness (lift-off). The maximum defect value measured is lower than 5 mm according to the conventional result line, and for the fuzzy output the value showed is almost 5 mm. The average defect at the sample plate ranges between 3.0 mm to 5.0 mm.

To validate the effectiveness of the proposed scheme, a comparison is made with (Yin & Xu, 2016) [29], where the percentage of error is shown in Table 4. The errors obtained at zero mm liftoff of coating thickness were 0.23% and 0.1% for Yin & Xu 2016 and the proposed method, respectively. The percentage of error can be calculated by following the equation

% of error = ((Actual Depth of Defect − Depth of Defect Measured)/(Actual Depth of Defect)) × 100%(3)

### 5.4. ANOVA Analysis Result

The two main parameters affecting defect measurement are frequency and lift-off. By following the SFECT system design, two input parameters are considered in measuring.

**Figure 14:**
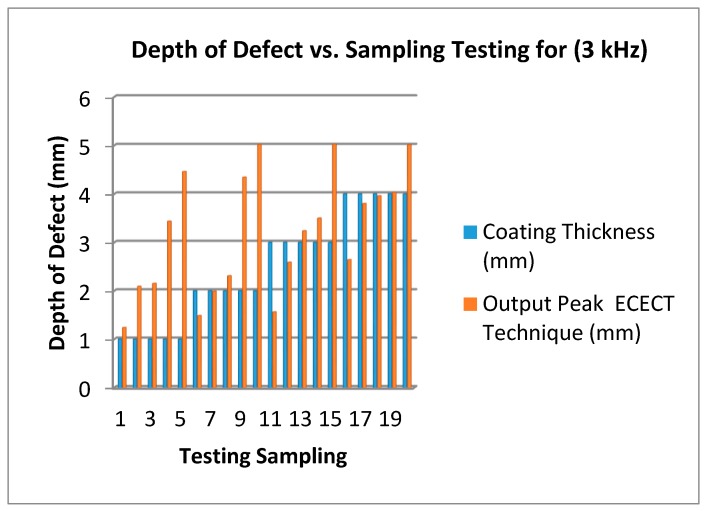
Depth of Defect (mm) vs. Testing Sampling (3 kHz Frequency).

Figure 14 shows the effect of the depth of the defect at different frequencies and coating thicknesses. The red bar color represents output peak and blue represents coating thickness. According to that bar graph, the accurate depth of the defect is shown when the defect plate is 5 mm and the output produced is 5 mm at coating thicknesses of 2 mm, 3 mm and 4 mm (at testing sampling 10, 15 and 20). The efficiency of the result using a 3 kHz frequency can reach more than 91% according to the tests done. This is because the frequency used can travel deeply within the surface plate defect, and the feedback from the signal is clear and accurate.

**Figure 15:**
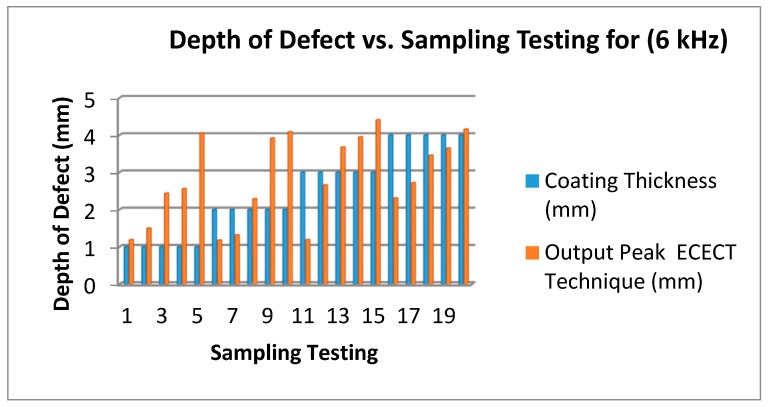
Depth of Defect (mm) vs. Testing Sampling (6 kHz Frequency).

**Figure 16:**
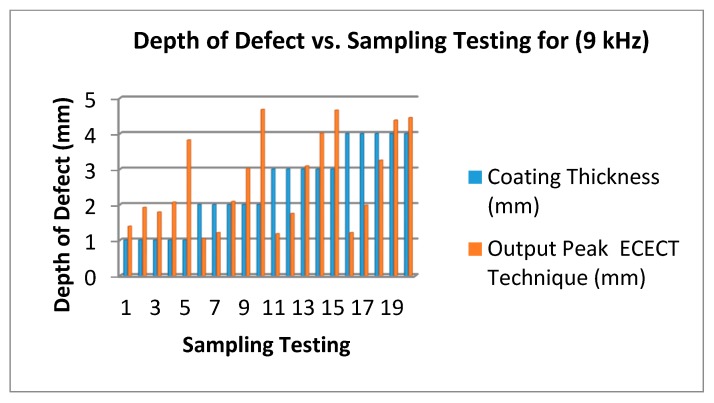
Depth of Defect (mm) vs. Testing Sampling (9 kHz Frequency).

On the second testing, a 6 kHz frequency is applied to the plate and the results are shown in Figure 15. The blue bar represents the coating thickness and red bar represents the SFECT output peak. Figure 16 shows that the accuracy is lower than 80% compared to using a 3 kHz frequency. The proposed probe is tested with a 5 mm depth of defect, and it displays a result range between 4.1 mm to 4.4 mm, which is only due to the lower frequency when traveling in plate testing; hence, the feedback or effect of the signal produced is lower too.

Lastly, Figure 16 shows the test carried out by using a 9 kHz frequency. According to the result shown, the accuracy of measurement is reduced by more than 74%. This can be seen for the test sampling at 5, 10, 15 and 20 where the lift-off is, respectively, 1 mm, 2 mm, 3 mm and 4 mm. The depth of the defect on the plate chosen is 5 mm. The resulting output measurements are 4.1 mm, 4.1 mm, 4.4 mm and 4.2 mm.

Figure 14, Figure 15 and Figure 16 show that the 3 kHz input excitation signal gives the best result in normal measuring. It was observed that the 1 mm coating thickness and 1 mm output peak gave the same result in all three frequencies (3 kHz, 6 kHz and 9 kHz) which in normal measuring are 1.24 mm, 1.19 mm and 1.39 mm, but the higher gap results could be obviously seen on a 4 mm coating thickness in a 2 mm output peak for all three frequencies: 3.8 mm, 2.27 mm and 1.98 mm, respectively.

Table 5 shows the response output peak result obtained from the design expert software simulation. Scientific analysis was done using the design expert software. Five parameters were analyzed: model, frequency, depth of defect, coating thickness and residuals. This analysis is based on the three frequencies used for inspection, namely 3 kHz, 6 kHz and 9 kHz. Besides that, the coating thickness on the sample measured is in the range of 1 mm to 4 mm. The crack depth at the coating plate is 1 mm to 5 mm.

*Y*_1_ = +0.32833 − 0.089417 × (*X*_1_ + 0.74817) × (*X*_2_ + 0.34180) × *X*_3_ (4)

*Y*_1_: Output Peak; *X*_1_: Frequency; *X*_2_: Depth Defect; *X*_3_: Coating Thickness.

**Figure 17:**
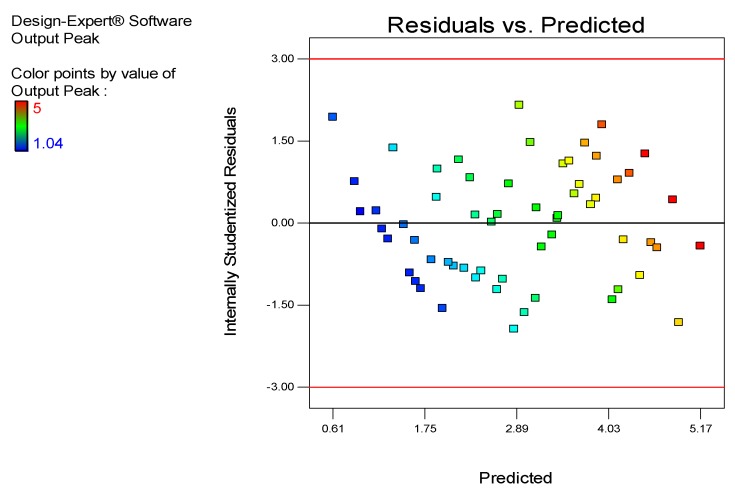
Residual vs. Predicted points.

Figure 17 shows the residual vs. predicted values. This figure shows the higher depth being measured in red, where the standardized residuals are in a range of −0.5 to 2.00, with the predicted range of 4.03 to 5.17 mm having 4 points. Every point shows the result of the testing based on sample testing considering the frequency, output, coating thickness and depth of defect. The lower output peak is shown at the point 0.16 of the predicted line and 2.00 of the residual area. The medium output peak mostly ranges between 1.75 mm to 4.03 mm in the predicted area and −1.50 to 2.00 in the residual area. The range of the residual point is set from −3.00 to 3.00, and the maximum output peak is 5, whereas the lower is 1.04.

**Figure 18:**
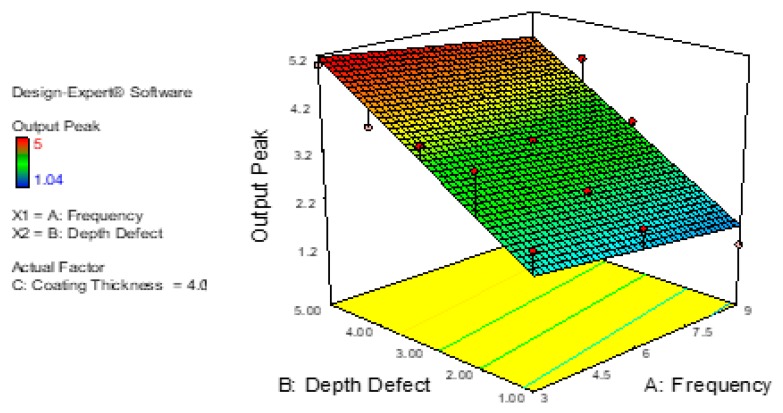
Relations between Depth of Defect, Frequency and Output Peak.

Figure 18 shows the frequency of the probes’ effect on the signal traveling on the plate tests. The maximum output result is 5.2 mm at the 3 kHz frequency used for the depth of defect point at 5.0 mm, whereas the lower value of the output peak, 1.2 mm, can be found at frequency 9 kHz at the depth of defect point 1.00 mm. The maximum output peak was observed in a range of 4.00 mm to 5.00 mm for a depth of defect at frequencies from 3 kHz to 6 kHz. Otherwise, the lower output peak can be defined at a range of 1.00 mm to 2.50 mm of the depth of defect at frequencies from 7.5 kHz to 9 kHz.

**Figure 19:**
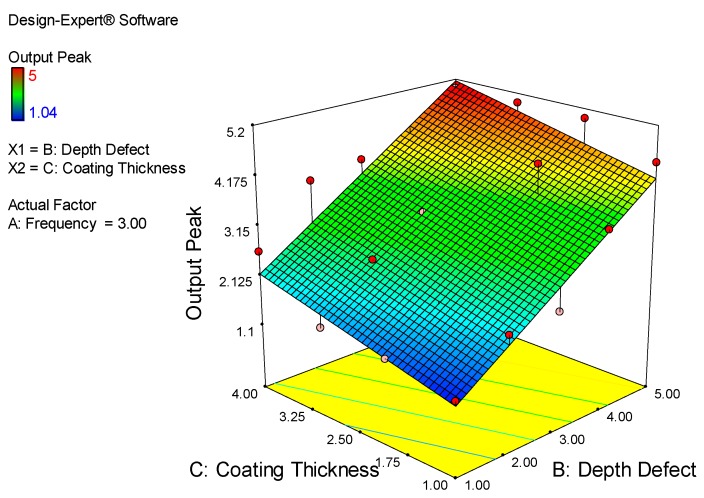
Relations between Depth of Defect, Coating Thickness and Output Peak.

The main factors that affect output peak are coating thickness and depth of defect, as shown in Figure 19. Most defects on the output peak are in the range of 3.15 mm to 4.5 mm and depth defects within 2.50 mm and 4.00 mm are shown in green, as per the graph below. From this graph, the dangerous situation or maximum crack (red color) was measured at 4.00 mm to 5.00 mm for depth of defect and at the coating thicknesses of 2.50 mm to 4.00 mm. The lower output measured is in the range of 1.00 mm to 2.00 mm in the depth of defect with a coating thickness of 1.00 mm to 2.50 mm.

**Figure 20:**
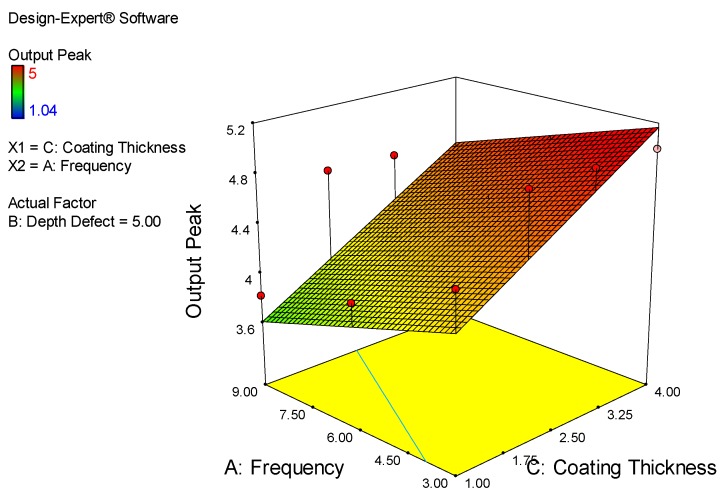
Relations between Frequency, Coating Thickness and Output Peak.

Lastly, the majority of the higher output peak was obtained according to Figure 20. It is observed that the range of coating thickness is 2.50 mm to 4.00 mm and the frequency is 3.00 mm to 6.00 kHz. The majority of the red color is in that area. The lower output peak being simulated is at point 7.50 mm to 9.00 mm for frequency and 1.00 mm to 1.75 mm for coating thickness.

## 6. Conclusions

In this paper, SFECT has been developed to solve the measurement error of the depth of defects that is caused by lift-off variations. An extensive literature review has been conducted in the early stage to reveal the lift-off error compensation method of differential and absolute probes. From there, it is found that the lift-off error compensation method has been given high attention due to its ability to improve the measurement accuracy of the sensor. The SFECT compensation method is divided into two types, which are hardware compensation and software compensation. In this paper, both compensation methods are used. In order to demonstrate the measurement error of the sensor under study caused by the lift-off variations, an experimental setup for data collection has been conducted. The inaccuracy and error analysis have been done, and a lift-off error compensation method has been proposed by using a Mamdani-type fuzzy inference system. The input MF was chosen according to the simplicity of the coding algorithm for hardware-based real-time implementation. Rules have been developed based on expert knowledge. The SFECT gives optimal correction for lift-off in which the reduced percentage error is only within 0.1% of its full-scale value. The SFECT is appropriate for measuring lift-off within a range of 1 mm to 5 mm.

## Figures and Tables

**Table 1:** Review of Lift-off Compensation Techniques.

Author	Technique Software or Hardware	Sensor Type	Research Area
[30]	Time domain analysis and frequency domain analysis based on differential responses	Pick-up coil is located orthogonally in the center at the bottom of the excitation coil	Reducing the lift-off problem and classify the defects.
[12]	Measuring the defect dimension based on the slope of the linear curve of the peak value	Hall sensor	Reducing the lift-off noise for detection of the defect depth or width
[31]	Hough transform was used	Coil	Investigating the lift-off effect in the normalized impedance plane
[32]	The theory of the linear transformer	GMR	Measuring the thickness of a metallic non-ferromagnetic plate
[33]	Normalisation technique	Coil	Minimise lift-off impact. It could be utilized to measure metal thickness and for microstructure analysis.
[34]	Analytical model that describes the inductance	Air-cored coil	The sensor phase signature analysis reveals that liftoff is independent for the testing plate.
[35]	Introducing a novel Permeability measurement approach	Coil	Investigating the phenomenon of conductivity invariance with a controlled lift-off for magnetic plates.

**Table 2:** Mamdani-Type Fuzzy Interface System (FIS) Rules.

Rules	Differential Probe	Absolute Probe	Depth of Defect
1	lowdefect	Lowliftoff	normaldefect
2	depthdefect	Lowliftoff	dangedefec
3	depthdefect	mediumliftoff	baddefect
4	depthdefect	highliftoff	baddefect
5	dangerdefect	lowliftoff	dangerdefect
6	dangerdefect	mediumliftoff	dangerdefect
7	dangerdefect	highliftoff	baddefect
8	lowdefect	mediumliftoff	baddefect
9	lowdefect	highliftoff	baddefect

**Table 3:** Differential and Absolute Probe Measuring.

Frequency (kHz)	Differential Probe (%)			Absolute Probe (%)			
	1 mm	2 mm	3 mm	0.5 mm	1 mm	1.5 mm	2 mm
4	2	5	10	20	10	5	2
10	10	20	25	65	40	20	5
20	20	60	80	100	65	45	15

**Table 4:** Comparison of liftoff with different coating thickness.

Liftoff (mm)	Error from (Yin & Xu, 2016) (%)	Error from ECECT (%)
zero	0.23	0.10
1.5	0.64	0.50
3	1.36	0.40
4.5	1.63	0.87

**Table 5:** Response Output Peak.

Source	Sum of Squares	df	Mean Square	*F* Value	*p*-Value Prob > *F*
Model	78.81	3	26.27	146.08	<0.0001
*A-Frequency*	2.88	1	2.88	16.01	0.0002
*B-Depth Defect*	67.17	1	67.17	373.51	<0.0001
*C-Coating Thickness*	8.76	1	8.76	48.72	<0.0001
Residual	10.07	56	0.18		
Cor Total	88.88	59			

Where df: It is the sum of the squares of the deviations from the means; *F* Value: is a value of the means between two populations are significantly different. *p* value: it is the indication of significance level.
